# Editorial: Shiga toxin-producing *Escherichia coli* (STEC) infections and consequences

**DOI:** 10.3389/fcimb.2022.1015653

**Published:** 2022-08-29

**Authors:** Jorge Goldstein, Leticia Bentancor

**Affiliations:** ^1^ Laboratorio de Neurofisiopatología, Instituto de Fisiología y Biofísica Houssay, Facultad de Medicina, Universidad de Buenos Airese, Buenos Aires, Argentina; ^2^ Consejo Nacional de Investigaciones Científicas y Técnicas, Buenos Aires, Argentina; ^3^ Instituto de Estudios para el Desarrollo Productivo y la Innovación, Universidad Nacional de José C. Paz, Buenos Aires, Argentina

**Keywords:** SOS response treatment, evolutionary genomic strain analysis, antibodies, cytokines, antibiotics, platelets, Neutrophil extracellular traps (NET), nitric oxide

Infection with Shiga toxin (Stx) producing *Escherichia coli* (STEC) causes hemorrhagic colitis; it is a serious public health problem. In some cases, it can evolve to Hemolytic Uremic Syndrome (HUS), an illness characterized by a triad of events including non-immune haemolytic anemia, thrombocytopenia, and acute renal failure caused by Shiga toxin (Stx) expressed by STEC (Fakhouri et al., 2017). Once Stx reaches the bloodstream it may target endothelial, kidney, and/or brain cells through the Stx globotriaosylceramide receptor (Gb3), causing cytotoxicity (Celi et al., 2022). Neurological impairment frequently occurs and is associated with a worse prognosis. In addition to Stx2 pathogenicity, lipopolysaccharide (LPS) is another virulence factor that is also released from STEC, enhancing the deleterious effects of Stx in different cells and organs (Goldstein et al., 2021).

The current Research Topic received six relevant works from worldwide. We will describe the more relevant aspects and conclusions about them.

## Mechanisms involve in Stx expression or damage regulation

First, we can mention the results published by Izquierdo et al.. Authors studied the role of two bacteria that are associated with infections with diarrheagenic *E. coli* (DEC). They analyzed *Citrobacter werkmanii* (CW) and *Escherichia albertii* (EA). The work analyzes the effects of supernatants of CW and EA on DEC. They observed that the supernatants provoke a modulation on gene expression of STEC and EAEC and they also reported an induction of IL-8 secretion by EA.

The next report was published by Eppinger et al.. They describe a clinical case of a laboratory worker who suffered an *E. coli* infection. This work showed and confirmed the importance of providing no treatment with antibiotics during STEC infection. They reported that the use of antibiotics in sublethal doses increased Stx expression and induced the development of HUS even in adults. In addition, this work puts in evidence the risks of simultaneous co-infection.

Landoni et al. describe the role of platelets (Plts) in neutrophil extracellular traps (NETs) induction and how it contributes to endothelial cell injury. They showed that Plts have a role as inducers of netosis. This netosis induction contributed to endothelial cell injury in cases of HUS. Those results have an implication on HUS treatment, the authors proposed that a modulation of Plts could be an option as treatment in order to control endothelial damage.

## SOS response treatment and evolutionary genomic strain analysis

Crane et al. demonstrated that the nitric oxide donor SNAP (S-nitroso-acetylpenicillamine) has the ability to inhibit the SOS response-leading antibiotic resistance in *E. coli*, in such a way that it significantly reduces the expression of the SOS marker RecA, involved in downstream Stx production and DNA hypermutation. Accordingly, this donor significantly reduced the expression of Stx at the protein and mRNA levels and DNA hypermutation particularly in STEC and EPEC strains (SOS induced-hypermutation causes these bacteria to be resistant to antibiotics like zidovudine and ciprofloxacin). In addition, the authors argue that combination of zinc acetate with SNAP could display an additive inhibitory activity against emergence of new antibiotic resistance. When the hypermutation is reduced by SNAP, pathogenic strains remain antibiotic sensitive. However, endogenous nitric oxide located in the intestinal mucosa does not seem to exert an inhibitory SOS effect under the performed assessments.

The work of Michelacci et al. shows a valuable genomic analysis of a very significant sample of strains obtained in Italy, from the last 30 years. In Italy, the predominant serogroup is STEC O26 among pediatric HUS cases since the 1990s. The impact of this study is that the authors managed to group these strains according to their virulence characteristics: Stx type identification, virulent genes within the LEE locus, and antibiotic resistant genes mainly in 2 large groups, ST21 and ST29. The HCPC analysis yielded 7 defined clusters. It is of interest that an association of characteristic patterns related to mobile genetic elements with specific clones has been found. This would have favored the occurrence and maintenance of an ecological reservoir. On the other hand, a low level of genomic variability has been observed, seen by clusters and clades, identified in ST29, which confirms the hypothesis that there was a selective pressure in specific ecological niches for STEC 026. This is not the case for ST21, which turned out to be more variable. ST29 appeared later, in the late 1990s.

## Antibodies against Stx: Update and perspectives

Finally, this issue updates in review format the use of antibodies developed against Shiga toxin for preventive use, byHenrique et al. A complete review of the antibodies developed against Shiga toxin in the last 30 years is provided, their trend is analyzed and what perspectives they would have as a therapeutic strategy. All types of antibody development were reviewed, from polyclonal, monoclonal antibody-secreting hybridoma, to recombinant antibody fragments as a therapy tool.

## Conclusions

The reports published in this Research Topic contribute to the understanding of different aspects implicated in Shiga Toxin-Producing Escherichia coli (STEC) Infections. As global conclusions we can highlight the following: antibiotics administration in patients with STEC infections provoke an increase in Stx production and worsen the clinical infection; the host gut microbiota could have a relevant role in the regulation of gene expression when diarrheagenic *E. coli* infection occurs; the aggregates of PMN-Plts or NETs promote the endothelial damage; proposed donors of nitric oxide that are used in the clinic such as the oral nitrovasodilators isosorbide mononitrate or isosorbide dinitrate, are suggested to be provided against SOS response; regarding the study of STEC 026, the greatest evolutionary variability of this strain occurred in plasmid pO26, which have stabilized and remained in specific niches in Italy; and finally, among other recommendations, for therapy with antibodies to neutralize the deleterious effect of the toxin, in addition to taking into account its immunogenic properties, the time of administration and the dose to be used should be considered.

We can conclude that this Research Topic contributed to new insights in relevant and multidisciplinary lines of research related to HUS.

## Author contributions

All authors listed have made a substantial, direct, and intellectual contribution to the work and approved it for publication.

## Acknowledgments

We want to acknowledge the selfless work of the participating reviewers and Frontiers staff who were involved in creating this special issue.

## Conflict of interest

The authors declare that the research was conducted in the absence of any commercial or financial relationships that could be construed as a potential conflict of interest.

## Publisher’s note

All claims expressed in this article are solely those of the authors and do not necessarily represent those of their affiliated organizations, or those of the publisher, the editors and the reviewers. Any product that may be evaluated in this article, or claim that may be made by its manufacturer, is not guaranteed or endorsed by the publisher.
